# Metastatic Soft Tissue Sarcomas in Adolescents and Young Adults: A Specialist Center Experience

**DOI:** 10.1089/jayao.2020.0010

**Published:** 2020-12-10

**Authors:** Eugenie Younger, Olga Husson, Bernice Asare, Charlotte Benson, Ian Judson, Aisha Miah, Shane Zaidi, Alison Dunlop, Omar Al-Muderis, Winan J. van Houdt, Robin L. Jones, Winette T.A. van der Graaf

**Affiliations:** ^1^ Sarcoma Unit, Royal Marsden NHS Foundation Trust, London, United Kingdom.; ^2^ Division of Clinical Studies, Institute of Cancer Research, London, United Kingdom.

**Keywords:** soft tissue sarcoma, metastatic, chemotherapy, outcomes

## Abstract

***Purpose:*** Soft tissue sarcomas (STS) account for 8% of all cancers in adolescents and young adults (AYAs). Metastatic STS contribute significantly to disease-related mortality in this age group; however, data are limited due to under-representation in clinical trials.

***Methods:*** AYAs aged 18–39 years, diagnosed with metastatic STS between 1990 and 2012, were identified from The Royal Marsden Hospital database. Outcomes of interest were clinical characteristics, treatment patterns, overall survival (OS), and prognostic factors.

***Results:*** Overall, 455 patients were included. Median age at diagnosis of metastatic STS was 33 years (interquartile range [IQR] 27–37 years). The most common histological subtypes were leiomyosarcoma (*n* = 68, 15%), synovial sarcoma (*n* = 68, 15%), Ewing sarcoma (*n* = 44, 10%), and rhabdomyosarcoma (*n* = 35, 8%). Treatments included systemic therapy (*n* = 395, 87%; median 2 lines [IQR 1–3]; clinical trial *n* = 93, 22%), radiotherapy (*n* = 297, 66%), and metastasectomy (*n* = 191, 43%). Median duration between last chemotherapy regimen and death was 4.6 months (IQR 2–10). Median OS was 19.2 months (95% confidence interval [CI] 15.8–22.2); 5-year OS was 16%. Of common subtypes, patients with rhabdomyosarcoma had the worst OS (8.8 months; 95% CI 7.9–11.4). Adverse prognostic factors included male gender, synchronous metastases, bone or liver metastases, first-line polychemotherapy, and no metastasectomy.

***Conclusions:*** Outcomes were variable; patients with supposed chemosensitive subtypes had particularly poor survival. The diverse behavior of STS in AYAs highlights the need for subtype-specific clinical trials.

## Introduction

Soft tissue sarcomas (STS) are rare heterogeneous tumors that can arise in patients of any age.^1^ Although STS account for 1%–2% of all adult cancers, STS are proportionally more common in adolescents and young adults (AYAs), comprising 8% of cancer diagnoses in this age group.^2^ Definitions of AYAs vary, however, the U.S. National Cancer Institute and European Network for Cancer in Children and Adolescents have accepted the range 15–39 years.^3^ Cancer is the leading cause of disease-related death in AYAs in high-income countries, and STS contribute substantially to mortality and loss of life years.^4^

The distribution of STS histological subtypes varies across the age spectrum. Rhabdomyosarcoma predominately occurs in children (typically embryonal subtype), however, can also arise in AYAs (more commonly alveolar subtype).^5^ Ewing sarcoma of the bone has a peak incidence in adolescence, whereas extraosseous Ewing sarcoma is more commonly seen in young adults.^6^ Synovial sarcoma, epithelioid sarcoma, and alveolar soft part sarcoma typically occur in young adults.^5^ STS classically affecting older adults, such as undifferentiated pleomorphic sarcoma (UPS), are also seen in patients in the upper age range of the AYA group.^5,7^

Many STS demonstrate an aggressive phenotype, and approximately half of all patients with initially localized (intermediate- or high-grade) STS will develop metastatic disease. Previous research has described the epidemiology, biology, and outcomes of AYAs with certain STS histological subtypes; however, data on the treatment and survival of AYAs with metastatic STS are limited.^3–15^ AYAs with sarcoma are under-represented in clinical trials due to multiple factors, including the separation between pediatric and adult care.^11^ Furthermore, when AYAs are included within clinical trials, subgroup analysis of their outcomes are not routinely performed and data are, not distinguishable from the whole trial population.^11^ Observational studies can, therefore, provide informative data on the natural history, treatment, and survival of AYAs with metastatic STS treated in clinical practice.^16^

The Royal Marsden Hospital (RMH) Sarcoma Unit is one of the largest STS units in Europe. The RMH Sarcoma Unit is led by adult oncologists, and predominately treats patients aged 18 years and older. Younger patients (aged <18 years) are treated in the Paediatric Unit, based at a different site, or referred to specialist units for teenagers and adolescents. The objectives of this study were to describe clinical characteristics, treatment patterns, prognostic factors, and clinical outcomes of AYAs, aged 18–39 years at diagnosis, treated for metastatic STS in the RMH Sarcoma Unit. These data will help guide the development of age-specific services, inform strategies for research, and assist the design of future clinical trials.

## Materials and Methods

AYAs aged 18–39 years diagnosed with STS and treated at the RMH for metastatic STS between January 1, 1990, and December 31, 2012, were identified from the prospectively maintained sarcoma unit database. Data collected from electronic patient records included patient characteristics (e.g. gender), date of diagnosis of STS, anatomical site and size of primary tumor, histological subtype and grading (Fédération Nationale des Centres de Lutte Contre le Cancer [FNCLCC] criteria), number and location of metastases at first diagnosis of metastatic disease.

All cases were reviewed by an expert STS pathologist. Molecular assays were chosen based on morphology and immunohistochemistry. Reverse-transcription–polymerase chain reaction (PCR), fluorescence *in situ* hybridization, and additional PCR tests for the family of tumors with *EWSR1-CREB1* and *EWSR1-ATF1* fusion transcripts were performed where appropriate.

Three time periods were defined according to year of diagnosis of metastatic STS: 1990–1997, 1998–2005, and 2006–2012. Synchronous disease was defined as metastases within 3 months of STS diagnosis. Metachronous disease was defined as metastases >3 months after STS diagnosis.

Treatment recorded included primary surgery, metastasectomy, radiotherapy, isolated limb perfusion, radiofrequency ablation, systemic therapies (chemotherapy, targeted drugs, endocrine therapies, and phase I drugs), and stem cell transplant. Systemic therapies received, number of treatment lines, and best radiological response (Response Evaluation Criteria In Solid Tumors [RECIST] criteria version 1.1 where available) were recorded. Date of death, or last follow-up, was defined at the cutoff date of October 1, 2017, to ensure 5 years of follow-up data.

### Statistics

The primary endpoint was median overall survival (OS), defined from date of diagnosis of metastatic STS to the date of death, and censored at last follow-up. Secondary endpoints were median OS according to time period of metastatic STS diagnosis (already defined), median OS according to histological subtype, and prognostic factors for OS. Additional measures included description of patient characteristics, treatment patterns, and systemic therapy response rates.

Categorical variables were summarized using frequencies and percentages, and continuous variables reported using median, interquartile range (IQR) and 95% confidence intervals (95% CIs). The effects of sociodemographic and clinical factors on OS were analyzed using the chi-squared (χ^2^) test. Multivariate Cox regression was used to identify significant prognostic factors for OS.

## Results

### Patient characteristics

**FIG. 1.**
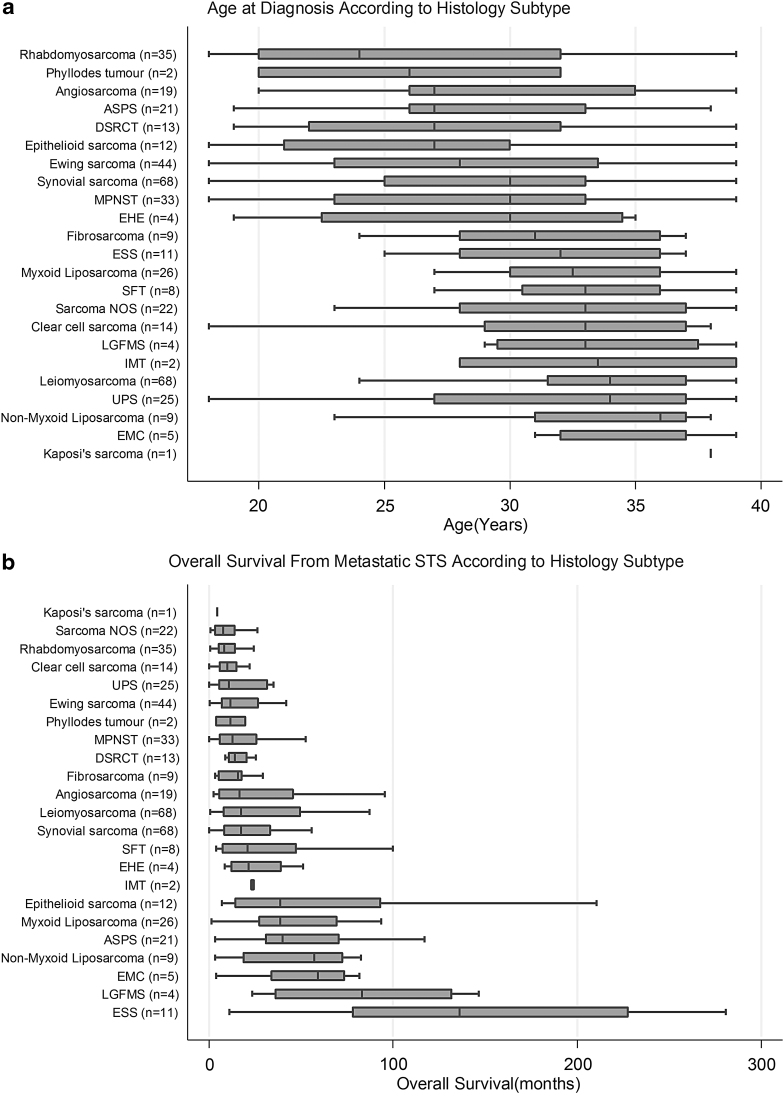
**(a)** Age at diagnosis of STS according to histology subtype. x-axis: Age (years). y-axis: Histological subtype (number of patients). **(b)** Overall survival from metastatic STS according to histology subtype. x-axis: Age (years). y-axis: Histological subtype (number of patients). ASPS, alveolar soft part sarcoma; DSRCT, desmoplastic small round cell tumor; EHE, epithelioid hemangioendothelioma; EMC, extraskeletal myxoid chondrosarcoma; ESS, endometrial stromal sarcoma; LGFMS, low-grade fibromyoid sarcoma; IMT, inflammatory myofibroblastic tumor; MPNST, malignant peripheral nerve sheath tumor; sarcoma NOS, sarcoma not otherwise specified; SFT, solitary fibrous tumor; STS, soft tissue sarcomas; UPS, undifferentiated pleomorphic sarcoma.

Four hundred fifty-five AYAs diagnosed with STS aged 18–39 years were included in the analysis (Table 1). Figure 1a demonstrates the median age at diagnosis according to STS histological subtype. One-third of patients presented with synchronous metastases and two-thirds developed metastatic disease after a median duration of 16 months (IQR 7–32). Median age at diagnosis of metastatic STS was 33 years (IQR 27–37 years). The extremity was the most common site of primary disease (*n* = 189, 42%) and median primary tumor size was 9 cm (range 0.6–57 cm). Half of STS were classified as histological grade 3 (*n* = 184, 53%). Patients with 22 histological subtypes were treated: highest frequency leiomyosarcoma (*n* = 68, 15%; uterine leiomyosarcoma: *n* = 20, “nonuterine” leiomyosarcoma: *n* = 48); synovial sarcoma (*n* = 68, 15%); Ewing sarcoma (*n* = 44, 10%); rhabdomyosarcoma (*n* = 35, 8%; alveolar: *n* = 22, embryonal: *n* = 9, pleomorphic/not otherwise specified (NOS): *n* = 4); liposarcoma (*n* = 35, 8%; myxoid *n* = 26, “nonmyxoid” *n* = 9); malignant peripheral nerve sheath tumor (MPNST; *n* = 33, 7%); and UPS (formerly MFH; *n* = 25, 5%). At first metastatic diagnosis, most patients had one site of distant spread (*n* = 306, 67%), commonly pulmonary metastases (*n* = 268, 59%).

**Table 1.** Patient Characteristics

Variable	N (%)
Gender	
Male	233 (51)
Female	222 (49)
Site of primary tumor	
Extremity	189 (42)
Retroperitoneum	70 (15)
Intra-abdominal and pelvic	43 (9)
Gynecological	36 (8)
Intrathoracic	37 (8)
Head and neck	30 (7)
Other	50 (11)
Tumor grade	
1	58 (13)
2	103 (23)
3	184 (40)
Missing	110 (24)
Histology	
Leiomyosarcoma	68 (15)
Synovial sarcoma	68 (15)
Ewing sarcoma	44 (10)
Rhabdomyosarcoma	35 (8)
Liposarcoma	35 (8)
MPNST	33 (7)
UPS	25 (5)
Other^a^	147 (32)
Metastases	
Synchronous	147 (32)
Metachronous	308 (68)
Number of sites of metastases^b^	
1	306 (67)
2	112 (25)
3+	37 (8)
Sites of metastatic disease	
Lung	268 (59)
Liver	51 (11)
Bone	74 (16)
Brain	10 (2)
Abdominal/pelvic	127 (28)
Other	110 (24)
Time period of diagnosis	
1 (1990–1997)	125 (28)
2 (1998–2005)	160 (35)
3 (2006–2012)	170 (37)

^a^ “Other” histological subtypes are demonstrated in Figure 1a and b.

^b^ Number of sites of metastases at first diagnosis of metastatic disease.

Seventeen patients (4%) had a history of a previous malignancy and 67 patients (15%) had a family history of cancer affecting a first or second degree relative. One patient had Li–Fraumeni syndrome. Details of family history were not available for two-thirds of patients (*n* = 301, 66%).

All patients with alveolar rhabdomyosarcoma and molecular analysis (*n* = 9) had the *PAX3-FOXO1* fusion gene (formerly *PAX3-FKHR*). Thirteen patients with MPNST (39%) had an *NF-1* mutation (20 patients with available data).

### Treatment patterns

Three hundred twenty-two patients (71%) had surgical resection of the primary tumor and 191 patients (42%) had at least one metastasectomy (Table 2 and Supplementary Table S1). Two-thirds of patients received radiotherapy (*n* = 297, 66%). The majority of patients were treated with systemic therapy (*n* = 395, 87%). Patients received a median of two lines of systemic therapy (range 1–8). Anthracycline-based chemotherapy was the most common first-line treatment (*n* = 211, 46%); doxorubicin plus ifosfamide (*n* = 86) or single agent doxorubicin (*n* = 82) (Table 3). Ninety-three patients (22%) participated in a clinical trial: 16 patients (4%) received a phase I or II trial drug as first-line systemic therapy and 14 patients (3%) took part in more than one clinical trial (Supplementary Table S2).

**Table 2.** Treatment Patterns and Overall Survival

	All patients (*n* = 455),* n *(%)	LMS (*n* = 68),* n *(%)	SS (*n* = 68),* n *(%)	EWING (*n* = 44),* n *(%)	RMS (*n* = 35),* n *(%)	LIPO (*n* = 35),* n *(%)	MPNST (*n* = 33),* n *(%)	UPS (*n* = 25),* n *(%)
Locoregional treatments								
Surgery (primary)	322 (71)	53 (78)	53 (78)	20 (46)	8 (24)	34 (97)	28 (85)	22 (88)
Metastasectomy	191 (42)	30 (46)	31 (46)	10 (23)	1 (3)	25 (71)	10 (30)	14 (56)
Radiotherapy	297 (66)	35 (53)	45 (66)	32 (73)	26 (79)	22 (63)	23 (70)	20 (80)
RFA	12 (3)	5 (7)	0 (0)	1 (2)	0 (0)	0 (0)	1 (3)	1 (4)
ILP	8 (2)	0 (0)	4 (6)	0 (0)	0 (0)	0 (0)	0 (0)	1 (4)
Systemic therapy	395 (87)	60 (88)	66 (97)	43 (98)	34 (97)	30 (86)	28 (85)	22 (88)
First line chemotherapy								
Anthracycline based	211 (58)	40 (70)	37 (64)	9 (24)	10 (35)	18 (51)	18 (69)	18 (90)
Polychemotherapy	187 (51)	32 (56)	30 (52)	29 (78)	24 (86)	4 (18)	12 (46)	12 (60)
Clinical trial								
Any phase	93 (22)	15 (23)	16 (27)	9 (24)	5 (16)	5 (14)	6 (20)	5 (22)
Phase I or II	54 (12)	8 (12)	9 (13)	5 (11)	0 (0)	5 (14)	3 (9)	2 (8)
Stem cell transplant	14 (3)	0 (0)	2 (3)	8 (18)	4 (11)	0 (0)	0 (0)	0 (0)
Best supportive care only	9 (2)	1 (1)	0 (0)	1 (2)	1 (3)	0 (0)	3 (9)	1 (4)
Median OS (95% CI), months	19.2 (15.8–22.2)	20.1 (14.0–31.8)	19.5 (14.3–28.9)	13.4 (8.9–25.2)	8.8 (7.9–11.4)	42.1 (28.4–64.4)	12.9 (9.1–22.8)	11.2 (7.9–22.0)

CI, confidence interval; Ewing, Ewing sarcoma; LIPO, liposarcoma; LMS, leiomyosarcoma; OS, overall survival; RMS, rhabdomyosarcoma; SS, synovial sarcoma.

**Table 3.** Systemic Therapy Received According to Treatment Line

Systemic therapy name	First-line (*n*)	Second-line (*n*)	Third-line (*n*)	Fourth-line (*n*)	Fifth-line (*n*)
Doxorubicin	82	16	5	3	0
Ifosfamide	30	46	12	4	3
Doxorubicin+ifosfamide	86	14	3	0	0
Trabectedin	6	25	22	13	3
Gemcitabine+docetaxel	10	11	9	6	2
Caelyx	7	7	7	5	2
VIDE	8	0	0	0	0
VAC	11	4	0	1	0
IVAD	10	4	1	0	0
IVA	3	5	0	0	0
Cisplatin+etoposide	9	9	2	0	0
Ifosfamide+etoposide	6	8	2	3	0
Etoposide	9	11	5	4	1
Pazopanib	2	6	6	4	1
Paclitaxel	9	3	0	0	0
Phase I or phase II drugs	16	28	12	8	1
Other doxorubicin/caelyx-based regimen	8	3	2	0	0
Other ifosfamide-based regimen	6	1	0	0	0
Other doxorubicin (or caelyx)+ifosfamide-based regimen	10	0	1	0	0
Other polychemotherapy regimen	20	11	12	2	1
Other single agent	13	17	11	5	4
Endocrine therapy	5	8	3	4	2
High dose with stem cell rescue	0	2	3	0	1

IVA, ifosfamide, vincristine, actinomycin D; IVAD, ifosfamide, vincristine, doxorubicin; VAC, vincristine, actinomycin D, cyclophosphamide; VIDE, vincristine, ifosfamide, doxorubicin, etoposide.

**FIG. 2.**
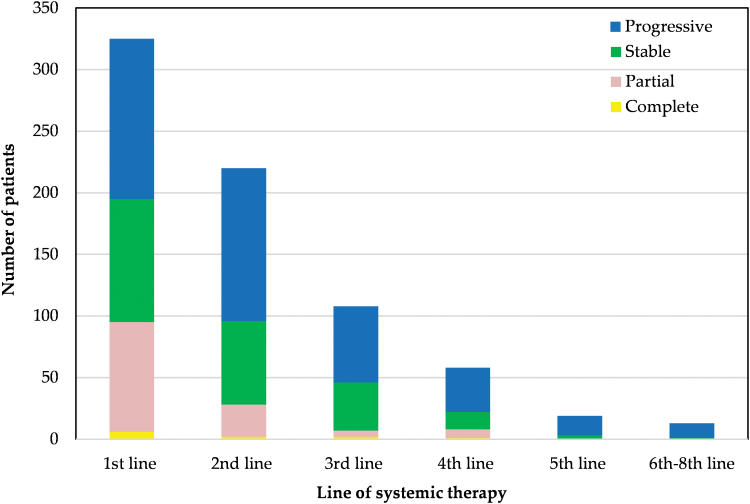
Best treatment response according to line of systemic treatment. x-axis: Line of systemic therapy. y-axis: Number of patients. Progressive disease—blue; stable disease—green; partial response—pink; complete response—yellow. Color images are available online.

The majority of radiological responses occurred in the first-line setting (complete response [CR] *n* = 6, partial response [PR] *n* = 89; overall response rate 27%) (Figure 2). The response rate to second-line treatment was 13% (CR: *n* = 2, PR *n* = 26). Treatment responses (CR or PR) beyond second-line treatment were uncommon (third line: *n* = 7, fourth line: *n* = 8, fifth line or beyond: *n* = 0). Around one-third of patients who received first-, second- and third-line systemic treatment had stable disease at best response (first line: *n* = 100, 31%; second line: *n* = 68, 31%; third line: *n* = 39, 36%).

The median duration between starting the last systemic treatment and death was 4.6 months (IQR 2–10): 32 patients started within 1 month of death (7%) and 119 patients started within 3 months of death (26%) (Table 4). Date of last chemotherapy cycle before death was available for 299 patients (66%): 62 patients received their last chemotherapy cycle within 30 days of death (21%) and a further 66 patients (22%) received their last chemotherapy cycle between 30 and 60 days of death.

**Table 4.** Duration Between Starting Last Systemic Therapy Regimen and Death

Time (months)	N (%)	Cumulative total (%)
Less than 1 month	32 (7)	32 (7)
1–1.99	49 (11)	81 (18)
2–2.99	38 (8)	119 (26)
3–3.99	28 (6)	147 (32)
4–4.99	18 (4)	165 (36)
>4.99	158 (35)	323 (71)

Percentages are expressed as proportion of whole patient group (*n* = 455).

### Overall survival

Median OS was 19.2 months (95% CI 15.8–22.0). The 5-year OS rate was 16%. There was no significant difference in median OS according to time period of diagnosis (*p* = 0.89).

Median OS for the most common STS subtypes: leiomyosarcoma 20.1 months (95% CI 14.0–31.8), uterine leiomyosarcoma 28.1 months (95% CI 15.0–82.3), “nonuterine” leiomyosarcoma 15.0 months (95% CI 11.3–31.8), synovial sarcoma 19.5 months (95% CI 14.3–28.9), Ewing sarcoma 13.4 (95% CI 8.9–25.2), rhabdomyosarcoma 8.8 months (95% CI 7.9–11.4), MPNST 12.9 months (95% CI 9.1–22.8), myxoid liposarcoma 40.3 months (95% CI 28.4–60.9), nonmyxoid liposarcoma 64.4 months (95% CI 3.5–82.7), UPS 11.2 months (95% CI 7.9–22.0), and “other” subtypes 21.6 months (95% CI 16.0–27.2). Figure 1b shows median OS according to all histological subtypes (Appendix Table A1 and Supplementary Table S3). Patients with history of a previous malignancy (*n* = 17) had a median OS of 8.6 months (95% CI 4.8–14.0).

On multivariate analysis (Table 5), adverse factors for OS were male gender (hazard ratio [HR] 1.4 [95% CI 1.1–1.8]), synchronous metastases at diagnosis (HR 1.9 [95% CI 1.4–2.5]), bone or liver metastases (bone: HR 1.7 [95% CI 1.2–2.4]; liver: HR 1.5 [95% CI 1.0–2.2]), polychemotherapy as first-line systemic treatment (HR 1.4 [95% CI 1.1–1.8]), no surgery for primary tumor (HR 1.4 [95% CI 1.0–1.9]), and no metastasectomy (HR 2.4 [95% CI 1.8–3.1]).

**Table 5.** Cox Multivariate Model

Covariate	p	HR (95% CI)
Gender		
Female		1
Male	0.003	1.41 (1.12–1.77)
Metastatic disease		
Metachronous		1
Synchronous	<0.001	1.88 (1.41–2.50)
Bone metastasis		
No		1
Yes	0.002	1.60 (1.18–2.17)
Liver metastasis		
No		1
Yes	0.031	1.48 (1.04–2.11)
Polychemotherapy first line		
No		1
Yes	0.004	1.42 (1.12–1.81)
Primary surgery		
Yes		1
No	0.043	1.39 (1.01–1.92)
Metastasectomy		
Yes		1
No	<0.001	2.35 (1.81–3.05)

HR, hazard ratio.

## Discussion

This study describes treatment patterns and clinical outcomes of a large group of AYAs with metastatic STS treated at a specialist sarcoma unit. OS was poor, although slightly better than the accepted prognosis for adult patients with metastatic STS treated in clinical practice (12–18 months).^17^ Patients with tumors regarded as chemosensitive, such as rhabdomyosarcoma and Ewing sarcoma, had particularly poor survival. Other histological subtypes demonstrated relatively indolent behavior. Multimodality treatment was common and systemic treatment was frequently started within the last 4 months of life, emphasizing the wish of patients or oncologists to treat, although a limited impact of palliative chemotherapy on survival.

Rhabdomyosarcomas in AYAs were most common of alveolar or embyronal “pediatric” subtypes. Although these tumors are typically sensitive to chemotherapy, survival was remarkably poor in these patients. Previous studies have also demonstrated that AYAs with rhabdomyosarcoma have poor outcomes compared with children.^18,19^ This has been attributed to unfavorable clinical features (e.g., alveolar subtype, nodal infiltration, and metastases at presentation), biological differences (e.g., multidrug resistance proteins), and undertreatment compared with intensive pediatric protocols.^5,19^

The majority of AYAs with rhabdomyosarcoma had an alveolar subtype (63%), and all of those with molecular analysis had expression of the *PAX3-FOXO1* fusion gene. Alveolar subtype and expression of the *PAX3-FOXO1* fusion gene are features associated with an aggressive phenotype and poor outcomes.^18^

AYAs often present with larger more invasive tumors due to late patient presentation and delayed recognition by health care professionals.^5,9^ Strategies to improve early diagnosis are not unjustified; however, the intrinsically aggressive behavior of rhabdomyosarcomas may prevail over any impact of diagnostic delay on survival.^20^ Where possible, patients at our institution received treatment within dose-dense protocols: predominately IVA (ifosfamide, vincristine, dactinomycin) or VAC (vincristine, dactinomycin, cyclophosphamide). Where dose intensity was reduced, this was due to tolerability or delayed bone marrow recovery rather than due to older age of the patients; inadequate treatment intensity was, therefore, not believed to be an important contributory factor for the poor outcomes of patients at RMH. Further understanding of underlying biology is needed to rationalize treatment protocols and develop more effective treatments.

Considering other common STS subtypes, AYAs with metastatic Ewing sarcoma, MPNST, and UPS had poor survival. Outcomes of AYAs with metastatic Ewing sarcoma (skeletal and extraskeletal) are acknowledged to be worse than for pediatric patients with the same histological subtypes; it is uncertain whether older age is an independent adverse prognostic factor, or whether older age carries a risk of other unfavorable factors such as primary metastatic disease and poor response to primary chemotherapy.^21–23^

More than a third of AYAs with MPNST had an *NF-1* mutation, which is associated with worse prognosis compared with sporadic MPNST.^10,24^ Patients with MPNST respond relatively poorly to chemotherapy and those arising in the setting of *NF-1* mutations may have inferior response rates.^25,26^ Loss of the NF-1 protein leads to activation of the RAS signaling pathway; however, therapeutic attempts to target RAS signaling and downstream pathways have had disappointing results.^27^ Clinical trials evaluating multiagent strategies (such as MEK and mTOR inhibitors) are ongoing and results are awaited.

Survival of AYAs with metastatic UPS was similar to that of adults with metastatic UPS treated in clinical practice, as reported by the French Sarcoma Group (AYAs: 10.8 months vs. adults: 11.2 months).^17^ Survival of AYAs with metastatic leiomyosarcoma was also in line with that of adults with metastatic leiomyosarcoma in the French Sarcoma Group study (AYAs: 20.1 months vs. adults: 19.4 months).^17^ Interpretation of a subgroup analysis of patients with uterine versus “nonuterine” leiomyosarcoma was limited by large CIs. Previous studies have reported no significant differences in the outcomes of patients with advanced or metastatic uterine leiomyosarcoma (uLMS) versus non-uLMS.^28–30^

Survival of AYAs with metastatic synovial sarcoma was similar to that of adults with synovial sarcoma in the French Sarcoma Group “METASARC” observational study (AYAs: 19.5 months vs. adults: 19.7 months).^31^ Others have reported a better prognosis for AYAs with metastatic synovial sarcoma compared with that for adults^.32,33^ Despite similar histological features, characteristic t(X;18) translocation, and fusion transcripts, adults with synovial sarcoma have worse outcomes compared to children with synovial sarcoma.^34^ Greater chromosomal instability in adults is associated with inferior metastatic outcomes; however, mechanisms leading to chromosomal complexity are not well understood.^35^ Furthermore, chromosomal instability does not predict response to chemotherapy.^36^ Future research may identify genomic alterations that are involved in response to treatment and could be targeted with novel or existing agents.^36^

The majority of AYAs with liposarcoma had a “myxoid” variant, characterized by a t(12:16)(q13;p11) translocation, resulting in the formation of a FUS-CHOP fusion oncoprotein (rarer aberrations include t(12;22)(q13;a12) resulting in DDIT3-EWSR1 fusion protein).^37^ AYAs with myxoid liposarcoma had a favorable outcome compared with many other STS subtypes, explained by the disease biology and sensitivity both to chemotherapy and radiotherapy. Myxoid liposarcomas are particularly sensitive to trabectedin, attributable to inhibition of gene transcription (affecting production of the aforementioned fusion oncoproteins), key cellular processes, and modulation of the tumor microenvironment.^38,39^ Trabectedin was not widely available during the study period; however, the pivotal randomized phase III trial of trabectedin versus dacarbazine for patients with metastatic liposarcoma or leiomyosarcoma (after failure of conventional chemotherapy) demonstrated that trabectedin is particularly effective in patients with liposarcomas (myxoid, pleomorphic, and dedifferentiated subtypes) and leiomyosarcomas.^40^ Trabectedin has also shown activity and clinical benefit in many other STS subtypes and it is probable the outcomes of AYAs with metastatic STS have improved since trabectedin was approved in the United Kingdom in 2010 (National Institute of Clinical Excellence).^41–43^

Consistent with previous studies, poor prognostic factors for survival included male gender, synchronous metastases at diagnosis, liver metastases, or bone metastases.^31,44,45^ Inferior outcomes for male patients may be due to faster clearance of the doxorubicin metabolite (doxorubicinol).^46^ First-line polychemotherapy was associated with poor survival, after adjusting for known prognostic factors. This contrasts with the METASARC study of adults with metastatic STS, which found that polychemotherapy was associated with better outcomes.^31^ It is probable that AYAs with intrinsically aggressive tumors were preferentially treated with polychemotherapy, and would have had a poor outcome irrespective of treatment.

Surgical resection of the primary tumor was associated with favorable outcome, and primarily represents patients with metachronous metastatic disease who had primary surgery before developing metastases. A small number of patients had palliative surgery to the primary site after developing metastases, such as limb amputation for intractable symptoms. Metastasectomy was also associated with improved survival after accounting for known prognostic factors. Better outcomes in these patients are likely to be attributable, in part, to the more indolent biological behavior of tumors that are selected for metastasectomy, and limited number of metastases in these cases (i.e., oligometastatic disease). The METASARC study reported similar rates of locoregional surgery in adults with metastatic STS (adults: 39% vs. AYAs: 42%).^31^

One-fifth of AYA patients participated in a clinical trial, which is slightly higher than overall rates of clinical participation in adults with cancer, estimated to be <5%.^11,47,48^ This may be because RMH is a tertiary referral center and the Sarcoma Unit is closely linked to the drug development unit (DDU). AYA patients may be more likely to participate in clinical trials at the DDU as they are less likely to have medical comorbidities that may preclude study entry. Patients who participated in a clinical trial as first-line systemic treatment for metastatic STS included patients who were treated within international phase III randomized trials, patients who entered phase I or II trials because no effective conventional treatments were available at that time (e.g., alveolar soft part sarcoma or clear cell sarcoma) or patients who had already received adjuvant chemotherapy with doxorubicin and ifosfamide. Patients who took part in clinical trials at later stages of their treatment trajectory (phase I or II trials) were those who had progressed through several lines of standard chemotherapy and had limited treatment options. Recently there have been several subtype-specific trials including AYA patients, such as a phase II trial with the EZH2 inhibitor (tazemetostat) for patients with advanced epithelioid sarcoma, and a placebo-controlled randomized phase II study with cediranib for patients with metastatic alveolar soft part sarcoma (CASPS).^49,50^

A large proportion of AYA patients received chemotherapy in the last months of life. High-intensity end-of-life care is common in AYAs with cancer, and inherent risks include hospitalization, intensive care unit admission, and intubation.^51–53^ Patients receiving active treatment are less likely to have palliative care involvement, which is essential for symptom control, advance care planning, and decisions surrounding stopping anticancer treatments. Very few patients received no active treatment and were managed with best supportive care; this contrasts with studies of older patients in whom frailty and comorbid conditions are important considerations.^31,54^

Very few radiological responses were seen beyond second-line systemic therapy; however, a third of patients attained stable disease with third-line treatment. Similarly, the METASARC study reported limited benefit of systemic therapy beyond the second-line setting (except leiomyosarcoma), with a median time to next treatment of 2–3 months.^31^ The absence of disease progression, not only tumor shrinkage, has been shown to have a favorable impact on disease control and survival.^55^ Research into the impact of disease stability on health-related quality of life may be valuable for patients making challenging treatment decisions toward the end of their life.

Patients with a history of previous malignancy had significantly worse OS compared with the overall group. This is consistent with the known adverse impact of a second primary malignancy on survival compared with a primary neoplasm in patients of the same age group.^56^ Younger age at diagnosis of STS is associated with a higher chance of inherited susceptibility; however, family history was not available for two-thirds of patients.^57^ Recognizing patients with inherited genetic defects can guide therapeutic decision making, screening and intervention (if at early stage), inform relatives of their own risk, and recommend investigations.^58^

### Limitations

Missing data were common, particularly for patients treated before the introduction of electronic patient records in 1997. Documentation of family history was limited; attention to this topic has become more prominent over the years with advances in genomic technology and development of systemic therapies that target specific genetic aberrations in patients with cancer.^58^ Selection bias was inherent in our patient sample; patients referred to RMH often have challenging disease and have exhausted standard treatment options at local centers. Time periods defined were arbitrary as they did not reflect any significant change in treatment paradigm.

## Conclusion

Survival of AYAs with metastatic STS varied according to histological subtype. Patients with tumors that are typically sensitive to chemotherapy had particularly poor outcomes. This demonstrates that tumor biology plays an important role in the outcomes of patients in this age group. Most patients had multimodal treatment and many received chemotherapy in the last few months of life, representing a high-intensity treatment approach. Further research into the underlying biological mechanisms and clinical trials that consider age and histological subtype are needed to improve outcomes.

## Supplementary Material

Supplemental data

## Appendix

**Appendix Table A1.** Overall Survival from Metastatic Soft Tissue Sarcomas According to Histological Subtype (Months)

Histology	N	Median	p25	p75	Min	Max
Kaposi's sarcoma	1	4.4	4.4	4.4	4.4	4.4
Sarcoma NOS	22	7.8	3.4	14.0	0.9	83.2
Rhabdomyosarcoma	35	8.3	5.2	14.2	0.5	135.9
Clear cell sarcoma	14	10.1	5.8	15.3	0.2	133.7
UPS	25	10.8	5.7	31.6	0.03	136.7
Ewing sarcoma	44	11.7	7.0	26.5	0.3	98.7
Phyllodes tumor	2	117.8	3.9	19.7	3.9	19.7
MPNST	33	13.7	5.8	26.1	0.2	130.6
DSRCT	13	14.2	11.0	20.5	8.9	101.4
Fibrosarcoma	9	15.7	5.4	18.1	3.5	112.9
Angiosarcoma	19	16.7	5.6	45.6	2.6	110.2
Leiomyosarcoma	68	17.4	8.2	49.6	0.5	250.7
Synovial sarcoma	68	17.5	8.5	33.5	0.2	201.0
SFT	8	21.0	7.7	47.6	3.9	100.0
EHE	4	21.5	12.2	39.1	8.6	51.1
IMT	2	23.6	23.0	24.2	22.9	24.3
Epitheloid sarcoma	12	38.6	14.5	93.3	7.0	216.1
Myxoid liposarcoma	26	38.8	27.4	69.4	1.3	270.0
ASPS	21	40.2	31.1	70.6	3.5	276.5
Nonmyxoid liposarcoma	9	57.3	18.7	72.5	3.4	222.3
EMC	5	59.2	33.9	73.4	4.1	81.7
LGFMS	4	83.2	36.3	132.0	23.7	146.5
ESS	11	136.0	78.7	227.8	11.2	280.8

ASPS, alveolar soft part sarcoma; DSRCT, desmoplastic small round cell tumor; EHE, epithelioid hemangioendothelioma; EMC, extraskeletal myxoid chondrosarcoma; ESS, endometrial stromal sarcoma; IMT, inflammatory myofibroblastic tumor; LGFMS, low-grade fibromyoid sarcoma; MPNST, malignant peripheral nerve sheath tumor; sarcoma NOS, sarcoma not otherwise specified; SFT, solitary fibrous tumor; UPS, undifferentiated pleomorphic sarcoma.
