# Supplementary material for: Metastatic Soft Tissue Sarcomas in Adolescents and Young Adults: A Specialist Center Experience
Source: J Adolesc Young Adult Oncol. 2020 Dec 10;9(6):628–38. doi: 10.1089/jayao.2020.0010 (PMC7757586; doi:10.1089/jayao.2020.0010)
Supplement: Supplemental data [file Supp_Tables1-3.pdf]

Supplementary Data

Supplementary Table S1. Number of Metastasectomies Received by Patients with Common Histological Subtypes

Histology	*1*	*2*	*3*	*4*	*>4*	*Unknown*
Leiomyosarcoma	20	6	2	1	1	2
Synovial sarcoma	20	7	4	0	0	0
Ewing sarcoma	7	1	0	1	0	1
Rhabdomyosarcoma	1	0	0	0	0	1
Liposarcoma	12	6	3	2	2	0
MPNST	5	5	0	0	0	0
UPS	8	1	1	1	3	0

MPNST, malignant peripheral nerve sheath tumor; UPS, undifferentiated pleomorphic sarcoma.

Supplementary Table S2. Number of Participants in Clinical Trials According to Line of Systemic Treatment

Line of systemic treatment	*Number of patients*
First	39
Second	35
Third	20
Fourth	10
Fifth	4
Sixth	1

Supplementary Table S3. Median Overall Survival in Patients with Common Histological Subtypes Treated with Doxorubicin or Doxorubicin Plus Ifosfamide

Histological subtype	*First line, doxorubicin (n)*	*First line, doxorubicin+ifosfamide (n)*	*First line doxorubicin, median OS (95% CI), months*	*First line doxorubicin+ifosfamide, median OS (95% CI), months*
Leiomyosarcoma	18	19	15.0 (6.2–31.8)	14.5 (11.3–65.0)
Synovial sarcoma	10	23	13.7 (4.7–35.5)	19.5 (10.7–35.1)
Liposarcoma	12	4	29.9 (25.6–NR)	40.3 (27.4–NR)
MPNST	7	9	5.8 (0.9–38.6)	26.1 (10.9–52.5)
UPS	6	11	8.6 (2.6–NR)	13.6 (7.9–35.1)

N.B. Ewing and rhabdomyosarcoma were not included due to their different treatment protocols.
